# Lightweight 3D Dense Autoencoder Network for Hyperspectral Remote Sensing Image Classification

**DOI:** 10.3390/s23208635

**Published:** 2023-10-22

**Authors:** Yang Bai, Xiyan Sun, Yuanfa Ji, Wentao Fu, Xiaoyu Duan

**Affiliations:** 1 Information and Communicaiton Schnool, Guilin University of Electronic Technology, Guilin 541004, China; by@guet.edu.cn (Y.B.); jiyuanfa@guet.edu.cn (Y.J.); fuwentaoaa@163.com (W.F.); duanxiaoyu@guet.edu.cn (X.D.); 2 Guangxi Key Laboratory of Precision Navigation Technology and Application, Guilin University of Electronic Technology, Guilin 541004, China; 3 National & Local Joint Engineering Research Center of Satellite Navigation Positioning and Location Service, Guilin 541004, China

**Keywords:** hyperspectral remote sensing image classification, deep learning, dense connection, stacked autoencoder

## Abstract

The lack of labeled training samples restricts the improvement of Hyperspectral Remote Sensing Image (HRSI) classification accuracy based on deep learning methods. In order to improve the HRSI classification accuracy when there are few training samples, a Lightweight 3D Dense Autoencoder Network (L3DDAN) is proposed. Structurally, the L3DDAN is designed as a stacked autoencoder which consists of an encoder and a decoder. The encoder is a hybrid combination of 3D convolutional operations and 3D dense block for extracting deep features from raw data. The decoder composed of 3D deconvolution operations is designed to reconstruct data. The L3DDAN is trained by unsupervised learning without labeled samples and supervised learning with a small number of labeled samples, successively. The network composed of the fine-tuned encoder and trained classifier is used for classification tasks. The extensive comparative experiments on three benchmark HRSI datasets demonstrate that the proposed framework with fewer trainable parameters can maintain superior performance to the other eight state-of-the-art algorithms when there are only a few training samples. The proposed L3DDAN can be applied to HRSI classification tasks, such as vegetation classification. Future work mainly focuses on training time reduction and applications on more real-world datasets.

## 1. Introduction

Hyperspectral Remote Sensing Image (HRSI) contains abundant spectral and spatial information of ground objects, so it is widely used in land use and land cover [1,2], forestry [3,4], precision agriculture [5,6], environmental monitoring [7,8], and military surveillance [9,10], etc. In these applications, the task of classification of HRSI is a universal and significant process, whose purpose is to identify the class of ground object for every pixel, because the classification accuracy determines the effect of applications. Unfortunately, HRSI not only provides rich spectral and spatial features of ground objects, but also contains a large amount of redundant information, which increases the difficulty of feature extraction and reduces the classification accuracy. This is the so-called curse of dimensionality. In addition, it is expensive to specify the class of ground object for each pixel manually, so insufficient labeled pixels accelerate the difficulty of feature extraction. The difficulty in improving HRSI classification accuracy with insufficient labeled samples restricts these applications. In order to extract effective features and improve classification accuracy, a large number of algorithms have been proposed.

Early methods mainly reduced the spectral dimensionality by hand-designed features. Band selection aims to select partial bands to replace original data for features extraction through certain criteria, such as semantic information of bands [11], kernel similarity of discriminative information [12], and affinity propagation algorithm by unsupervised learning [13]. Although band selection methods can effectively reduce dimensionality of bands, the directly removed bands may contain more or less important information for classification, resulting in reduced classification accuracy.

More dimensionality reduction methods are based on Feature Extraction (FE), which maps the original spectral bands to a new feature domain by some algorithms. Based on Principal Component Analysis (PCA), which is a linear unsupervised statistical transformation, the Tensor PCA (TPCA) [14], Joint Group Sparse PCA (JGSPCA) [15], and Superpixelwise Kernel PCA (SuperKPCA) [16] are proposed for HRSI classification in the spectral domain. Morphological Attribute Profiles (MAP) [17] is another feature extraction method widely used in HRSI classification. Ye et al. [18] employed PCA and extended multiple attribute profiles (EMAP) to extract features. Liu et al. [19] combined MAP and deep random forest for small sample HRSI classification. Yan et al. [20] improved the 2D singular spectral analysis (2DSSA) for extracting global and local spectral features by fusing PCA and folded PCA (FPCA). Traditional FE methods can only extract shallow features. This makes it difficult to further improve the classification accuracy.

In recent years, Deep Learning (DL) has achieved significant success in image processing due to its powerful capabilities of deep feature extraction. Researchers are inspired to introduce DL methods into HRSI classification tasks and have achieved better classification results than traditional methods. Chen et al. [21] proposed a 1D autoencoder network to extract spatial features and spectral features, respectively. Mario et al. [22] employed 1D Stacked Autoencoders (SAE) with three layers of encoder and decoder for pixel-based classification. Bai et al. [23] proposed a two-stage multi-dimensional convolutional SAE for HRSI classification, which was composed of the SAE-1 sub-model based on 1D Convolutional Neural Network (CNN) and the SAE-2 sub-model based on 2D and 3D convolution operations. Zhao et al. [24] proposed a deep learning architecture by combining an SAE and 3D Deep Residual Network (3DDRN), where the SAE is designed for dimensionality reduction and the 3DDRN is used for extracting spatial–spectral joint features. Cheng et al. [25] proposed a Deep Two-stage Convolutional Sparse Coding Network (DTCSCNet) for HRSI classification without back propagation and a fine-tuning process. Although the models based on SAE can be trained without any labeled samples, the existence of the decoder restricts the increase in the number of layers in the encoder, as overfitting easily occurs when the depth of the SAE is too large. This constrains the improvement of feature extraction ability.

CNN is the most widely used model in HRSI classification [26]. Jacopo et al. [27] made a shallow 1D-CNN with only one hidden layer to achieve state-of-the-art performance by label-based data augmentation. Li et al. [28] proposed a DCNR composed of a deep cube 1D-CNN and a random forest classifier for extracting spectral and spatial information. From the perspective of kernel structure, 1D-CNNs are only suitable for extracting spectral features. If they are used to extract spatial features, the 2D spatial tensors must be converted into 1D vectors and this transformation will result in the loss of spatial information. In order to fully utilize the spatial information, 2D-CNNs are introduced for HRSI classification. Haque et al. [29] proposed multi-scale CNNs with three different sizes of 2D convolutional kernels to extract spectral and spatial features. Jia et al. [30] proposed an end-to-end deep 2D-CNN based on U-net which took the entire HSI as input instead of pixel patches. In 2D-CNNs, the spatial and spectral information are extracted separately. This ignores the fact that there is related information between spatial and spectral features. Gao et al. [31] proposed a lightweight spatial–spectral network which was composed of a 3D Multi-group Feature Extraction Module (MGFM) based on 3D-CNN and a 2D MGFM based on Depthwise Separable Convolution. Yu et al. [32] proposed a lightweight 2D-3D-CNN by combining 2D and 3D convolutional layers, which can extract the fused spatial and spectral features. Hueseyin et al. [33] proposed a 3D-CNN based on LeNet-5 model to extract spatial and spectral features from data processed by the PCA method. Due to the structural consistency between 3D convolutional kernels and 3D-cube HRSIs, 3D-CNNs can effectively extract spatial–spectral joint features. Unfortunately, the excellent feature extraction ability of 3D-CNNs requires sufficient labeled samples for training, but there is a high cost of labeling sample results in that there are insufficient labeled samples for training.

Recently, how to improve the feature extraction ability of 3D-CNNs with a small training sample size has become a research hotspot. Li et al. [34] proposed MMFN based on 3D-CNN, in which multi-scale architecture and residual blocks are introduced to fuse related information among different scale features and extract more discriminative features. Zhou et al. [35] proposed a Shallow-to-Deep Feature Enhancement (SDFE) model, which was composed of PCA, a shallow 3D-CNN (SSSFE), a channel attention residual 2D-CNN, and a Vision-Transformer network. Ma et al. [36] proposed a Multi-level Feature extraction Block (MFB) for spatial–spectral feature extraction and a spatial multi-scale interactive attention (SMIA) module for spatial feature enhancement. Paoletti et al. [37] proposed a pyramidal residual module architecture, which is used to build deep pyramidal residual networks for HRSI classification. In addition to residual structure, attention mechanism is also widely used to improve the feature extraction ability of CNNs. Zhu et al. [38] proposed a Residual Spectral–Spatial Attention Network (RSSAN) for HRSI classification. In this model, the raw data are sequentially processed through a spectral attention module, a spatial attention module, and a residual block. In the Cooperative Spectral–Spatial Attention Network (CS2ADN) [39], the spectral and spatial features are extracted by the independent spectral and spatial attention branches, respectively. Then, the fused features are further extracted by dense connection structure. Generally, there are two residual branches in a model, which are named the spatial residual branch and the spectral residual branch. Similarly, there are two attention branches named the spatial attention branch and the spectral attention branch, respectively, in a model based on attention mechanism. This structure composed of two independent branches cannot extract spatial–spectral joint features and increases the number of trainable parameters in the model. When the number of labeled samples is small, the trainable parameters of models cannot be fully trained and the classification accuracy will decrease.

To improve the HRSI classification accuracy-based deep learning models under a small number of labeled samples, a model with a small number of trainable parameters and robust deep feature extraction capability is necessary. Inspired by these studies, a Lightweight 3D Dense Autoencoder Network (L3DDAN) is proposed in this paper for HRSI classification. From the top-level architecture, the network is an SAE composed of an encoder for extracting features and a decoder for reconstructing data. First, the SAE is trained without any labeled samples through unsupervised learning. Then, all labeled samples are randomly divided into a training group, validating group and testing group. The fine-tuned encoder and trained classifier are completed with a small number of training groups and validating groups by supervised learning. Finally, the classification ability of the trained classifier is evaluated with the testing group. The experimental results indicate that the L3DDAN can extract deep spatial–spectral features with only a small number of labeled samples. Thanks to this, the high classification accuracy can still be achieved when there are insufficient labeled samples. The major contributions of this paper include the following:

(1) A Lightweight 3D Dense Autoencoder Network (L3DDAN) is proposed for HRSI classification. The architecture of L3DDAN is an SAE based on 3D convolution operations and the Spectral–Spatial Joint Dense Block (S^2^DB) is introduced into the encoder to enhance the deep feature extraction ability. The high classification accuracy can be maintained when the number of training samples is small.
(2) An SAE architecture is proposed to train the encoder by unsupervised learning. The encoder of the SAE composes 3D convolution operations and S^2^DB to extract deep features from HRSI. The decoder is implemented by 3D convolution operations. The SAE enables the L3DDAN to extract deep features from origin data without labeled samples.
(3) The Spectral–Spatial Joint Dense Block (S^2^DB) is proposed to replace the traditional separated spatial residual branch and spectral residual branch. The S^2^DB not only avoids the loss of spectral–spatial joint features, but also reduces the number of trainable parameters in L3DDAN.

The rest of this paper is organized as follows. In Section 2, the detailed framework and related principle of L3DDAN are illustrated. In Section 3, the details and results of extensive experiments are provided. Finally, the conclusion is presented in Section 4.

## 2. Methodology

### 2.1. 3D Convolution Operation

**Figure 1:**
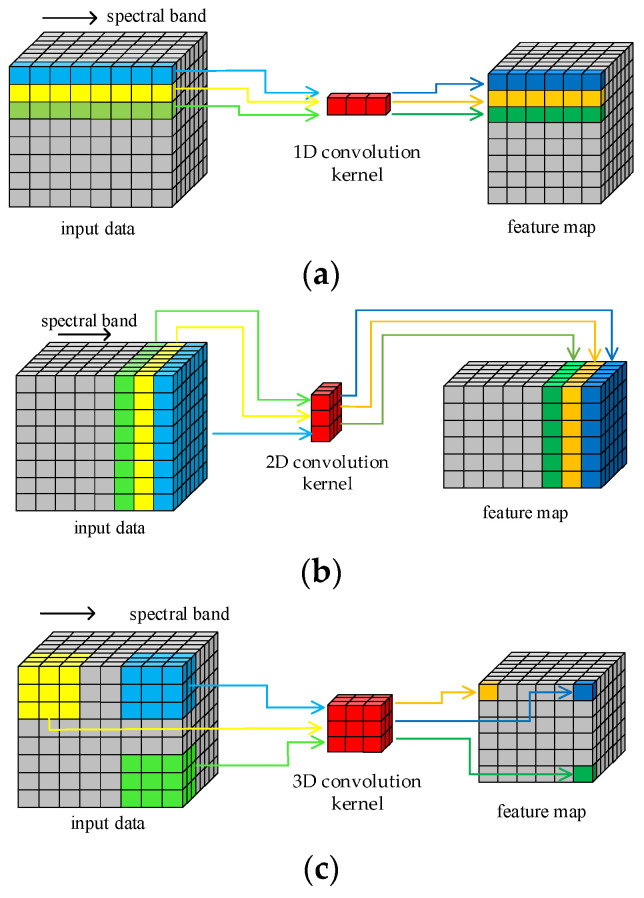
Convolution operations: (**a**) 1D; (**b**) 2D; (**c**) 3D.

The structure of HRSI data is a 3D cube that contains both spatial and spectral features. Figure 1 illustrates the principle of HRSI feature extraction by 1D, 2D, and 3D convolution operations, respectively. The 1D kernels in 1D-CNN can only extract spectral features and cannot extract spatial features. Similarly, only the spatial information in HRSIs can be extracted by the 2D convolution kernels. Only the 3D convolution kernels can extract both spatial and spectral features simultaneously, as they are structurally consistent with the 3D cube HRSI data. In order to improve the feature extraction ability of proposed model in this paper, all required convolution operations are implemented by 3D convolution kernels. This design enables the traditional separated spatial and spectral feature extraction branches to be replaced by just one block.

The 3D convolution operation can be formulated as follows:

(1) $$v_{i,j}^{x,y,z}=f\left(\sum_{m}\sum_{h=0}^{H_{i}-1}\sum_{w=0}^{W_{i}-1}\sum_{s=0}^{S_{i}-1}k_{i,j,m}^{h,w,s}v_{\left(i-1\right),m}^{\left(x+h\right)\left(y+w\right)\left(z+s\right)}+b_{i,j}\right)$$

where $v_{i,j}^{x,y,z}$ indicates the activation value at position $\left(x,y,z\right)$ in the jth feature map in the ith layer, m is the index of feature maps in the $\left(i-1\right)$th layer, $k_{i,j,m}^{h,w,s}$ is the value of the kernel at position $\left(h,w,s\right)$ connected to the mth feature map in the preceding layer, b is the bias and $f\left(\cdot \right)$ is the activation function, the H, W and S is the height, width, and spectral dimension size of the kernel, respectively.

In the L3DDAN, all convolution operations are implemented by 3D convolution kernels to avoid the loss of spatial–spectral joint features as much as possible. In addition, 3D convolution operations are used for spectral dimensionality reduction. This design makes the hyperspectral data without any preprocess to be directly used as the input of L3DDAN.

### 2.2. Spectral–Spatial Joint Dense Block

**Figure 2:**
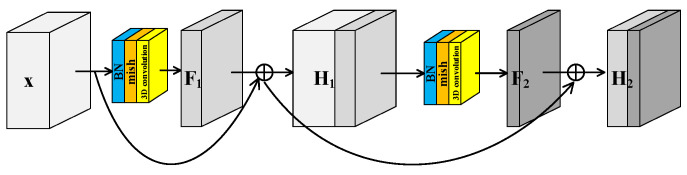
Spectral–spatial joint residual block.

He et al. [40] proposed the residual structure to solve the degradation problem of deep neural networks and make feature extraction easier without adding any learnable parameters. Many models [41] for HRSI classification have been proposed inspired by residual block. Generally there are two independent residual blocks in these models to extract spectral and spatial features, respectively. This structure of two separated branches not only fails to extract spectral–spatial joint features, but also increases the number of trainable parameters. In this paper, a Spectral–Spatial Joint Residual Block (S^2^RB) based on 3D convolution operations was proposed. In the S^2^RB shown in Figure 2, the input hyperspectral data cube $x\in R^{N\times W\times H}$ extracts features by 3D convolution operations to form output feature map $F(x)\in R^{N\times W\times H^{\prime }}$. The spatial dimensions of x and $F\left(x\right)$ are equal, and the spectral dimension is reduced from H to $H^{\prime }$. The introduction of a skip connection changes the mapping from $F\left(x\right)$ to $H\left(x\right)=F\left(x\right)+x$.

**Figure 3:**
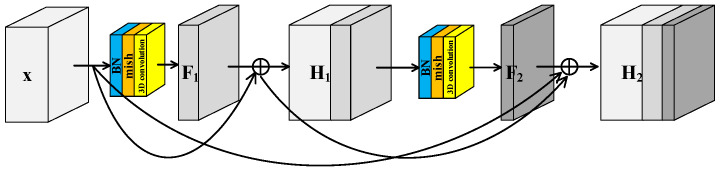
Spectral–spatial joint dense block.

To further improve the feature extraction ability of model, a Spectral–Spatial Joint Dense Block (S^2^DB) is proposed inspired by Dense Convolutional Network [42]. Different from S^2^RB, there are skip connections between each layer and all preceding layers in S^2^DB as shown in Figure 3. Consequently, the output feature maps of the first layer are formulated as $H_{l}=F_{1}+x$. This is the same as the output feature maps of S^2^RB. But for the second layer, the output feature maps of S^2^DB are formulated as $H_{2}=F_{2}+F_{1}+x$. Compared to the ***H_2_*** of S^2^RB, there is an additional term **x** in the ***H_2_*** of S^2^DB.

Previous models typically are composed of two separate branches for extracting spectral and spatial features, respectively. Because both S^2^RB and S^2^DB can extract spatial–spectral joint features, single branch structure can be adopted in the L3DDAN.

### 2.3. Proposed Framework

**Figure 4:**
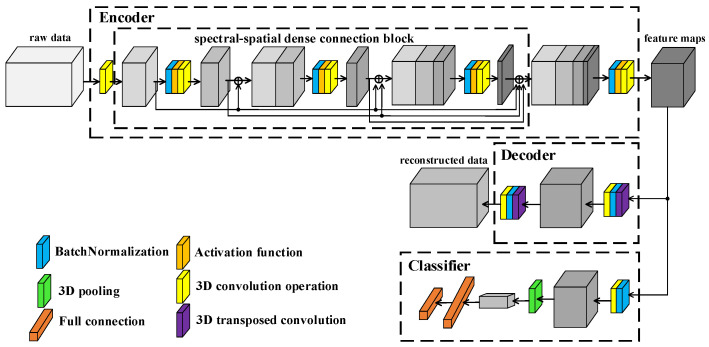
Framework of L3DDAN.

The framework of proposed L3DDAN is shown in Figure 4. Structurally, the model consists of an encoder, decoder, and classifier. The training and testing process is as follows: (1) The SAE composed of the encoder and decoder is first trained by unsupervised learning without any labeled samples. The purposes of encoder and decoder are to extract deep features from unpreprocessed data and reconstruct input data according to the feature maps, respectively. (2) After the training of SAE, the output feature maps from encoder are fed into the classifier. The fine-tuning for trained encoder and the training for classifier are completed simultaneously through supervised learning with small number of labeled samples. The decoder, which function is only to complete the training of the encoder, is not involved in this step. (3) The model composed of encoder and classifier can perform classification tasks on the test dataset.

The encoder of SAE is composed of 3D convolution operations and S^2^DB. The input data of L3DDAN are the patches of raw data without any preprocess. The spectral dimensionality of input data is reduced by the first 3D convolution layer. The S^2^DB is used to extract spatial–spectral joint features from the output data of previous layer. The deep features of raw data are obtained from the output feature maps of S^2^DB by the final 3D convolution layer of encoder. The output features maps of encoder are reconstructed by the decoder of SAE which composes two 3D deconvolution layers. In the classifier, the feature maps from encoder are classified by full connection layer after the last 3D convolution operation. Table 1 lists the detailed structure of the L3DDAN.

## 3. Results and Discussion

### 3.1. Datasets Description

**Figure 5:**
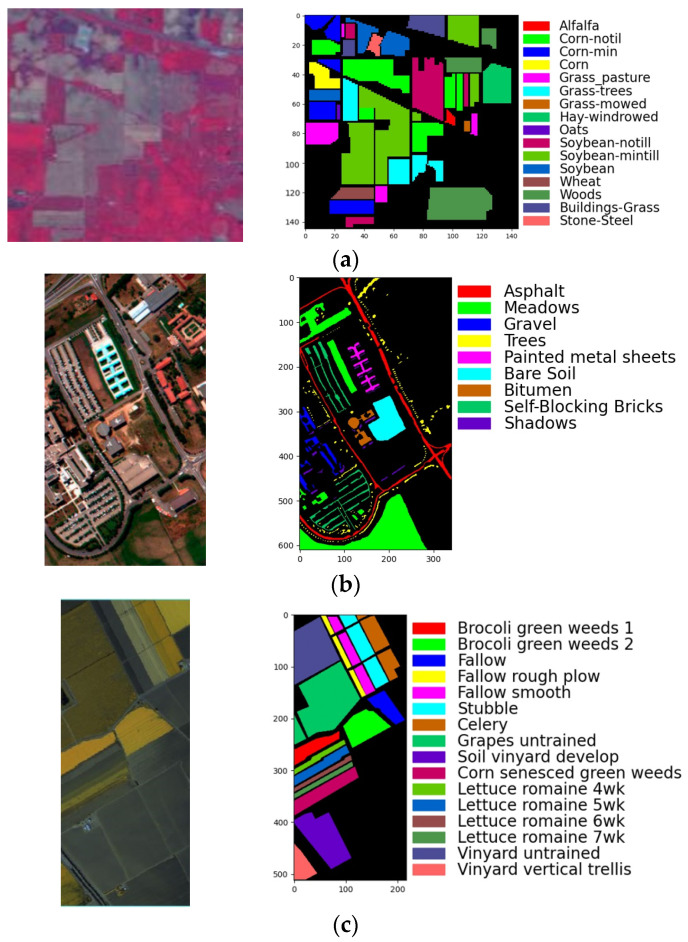
The pseudocolor images and ground-truth: (**a**) IP; (**b**) UP; (**c**) SV.

Three benchmark datasets were selected to validate the performance of the proposed L3DDAN for HRSI classification. The first dataset Indian Pines (IP) was acquired by the Airborne Visible Infrared Imaging Spectrometer (AVIRIS) instrument over a mixed vegetation site in northwestern Indiana, USA. It contains 16 cover types and 145×145 pixels with 220 bands covering from 0.4 μm to 2.5 μm. The noise-affected 20 bands (104–108, 150–163, 220) were removed and the retaining 200 bands were utilized for experiments. The labeled pixels of IP are randomly divided into training (3%), validation (3%), and testing (94%) groups. The second dataset University of Pavia (UP) was gathered by the Reflective Optics System Imaging Spectrometer (ROSIS) over the urban area of Pavia, northern Italy. It contains 610×340 pixels with 1.3 m spatial resolution and 103 spectral bands covering from 0.4 μm to 1 μm. All labeled pixels were divided into nine cover types. The third dataset Salinas Valley (SV) was captured over the Salinas Valley, CA, USA. It contains 512×217 pixels with 3.7 m/pixel spatial resolution and 204 bands in the wavelength ranging from 0.4 μm to 2.5 μm. The labeled pixels of UP and KSC are randomly divided into training (1%), validation (1%), and testing (98%) groups. The minimum number of samples per group is three for three datasets. The pseudocolor images and the ground-truth maps of three datasets are shown in Figure 5. The categories and pixel count for each dataset are listed in Table 2, Table 3 and Table 4.

### 3.2. Parameter Analysis

In the stage of SAE training, all samples were used for unsupervised learning without any labels. Then, all labeled samples are randomly divided into training group, validation group, and testing group. The training group was used for fine-tuning of encoder and training of classifier. The validation and testing groups were used to monitor the training process and evaluate the classification performance of L3DDAN, respectively. In the experiments, the overall accuracy (OA), average accuracy (AA), and Kappa coefficient [43] are used to quantitatively evaluate the classification performance. The proposed model was implemented by the Pytorch 1.10 framework (open source). All experiments were conducted on a PC (Lenovo, Shanghai, China) with Intel(R) Core i7-CPU, Nvidia Geforce GTX 3090 GPU and 64 GB RAM.

#### 3.2.1. Effect Analysis of the S^2^DB

**Figure 6:**
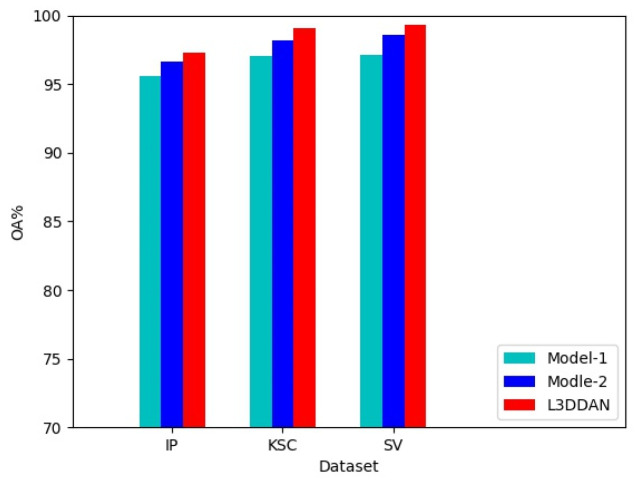
Effectiveness experiment of the S^2^DB.

To evaluate the effectiveness of the S^2^DB in L3DDAN, two comparative networks named Model-1 and Model-2 were constructed. The Model-1 and Model-2 networks were constructed by replacing the S^2^DB in L3DDAN with normal 3D convolution operations and S^2^RB, respectively. The three networks are identical except for the above difference. Comparative experiments were conducted under identical parameters and the experimental results are shown in Figure 6. It can be seen that the highest classification accuracies were achieved on all three datasets by L3DDAN, followed by the Model-2. This indicates that the dense connection structure further improves the feature extraction ability of the network compared to residual skip connection.

#### 3.2.2. Effect of the Patch Size

**Figure 7:**
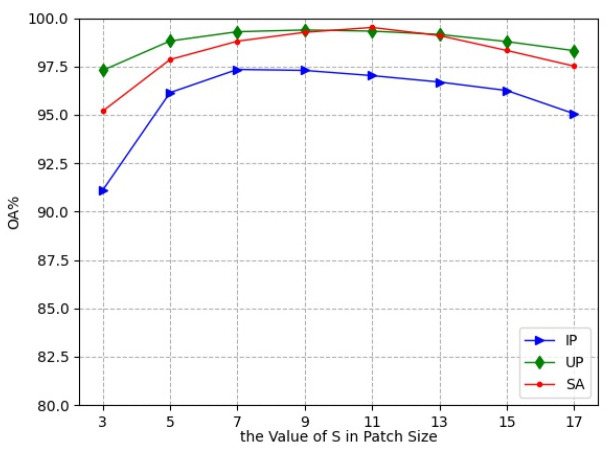
Classification accuracies with different patch size.

In the experiments, the small 3D neighboring patches $P\in R^{S\times S\times L}$ divided from HRSI data cube were used as the input of L3DDAN. The S is the number of pixels in spatial dimension and the L is the number of spectral bands. In this section, a series of comparative experiments were conducted to determine the value of S. The experimental results are shown in Figure 7.

As the spatial size increases from 3 to 17, the classification accuracies first increase and then decrease. The OA reached the maximum value when the spatial size of patches was 7, 9, and 11 for the IP, UP, and SA datasets, respectively. This indicates that increasing the patch size appropriately can bring more spatial information, but oversize introduces noise and reduces the classification accuracy. The patch sizes 7×7×L, 9×9×L, and 11×11×L were the optimal selections for the three datasets in the proposed network.

#### 3.2.3. Impact of the Number of Training Epochs

**Figure 8:**
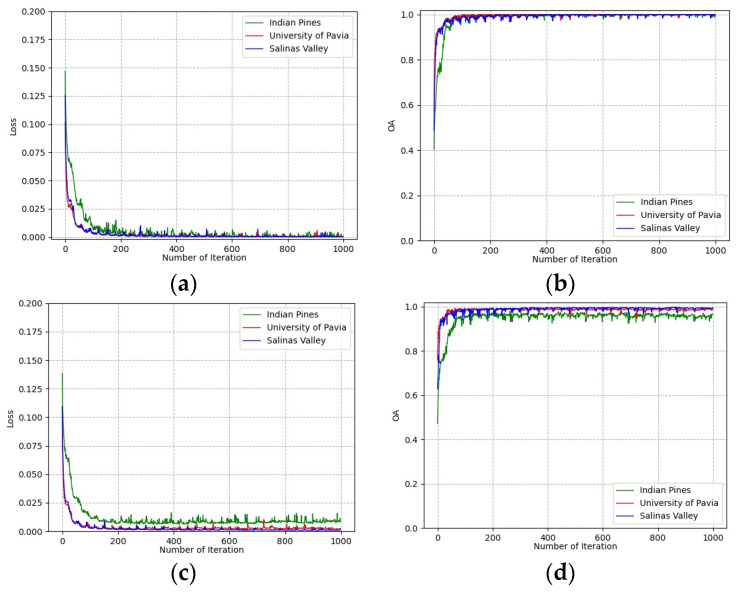
Loss and accuracy convergence versus epochs: (**a**) loss of training group; (**b**) accuracies of training group; (**c**) loss of validation group; (**d**) accuracies of validation group.

To determine the number of training epochs, the loss and classification accuracies of training and validation groups vary with the number of training epochs on all three datasets shown in Figure 8. It can be seen that all curves converge after 500 epochs on the training group and validation group. This indicates that the deep features extracted from the training group are effective for the classification of the validation group. The number of training epochs was determined to be 500 for all experiments.

### 3.3. Performance Evaluation

#### 3.3.1. Comparison of Classification Results

In this section, the proposed L3DDAN was compared with eight state-of-the-art algorithms including 1D-CNN [44], 2D-CNN [45], 3D-CNN [46], 3D-CAE [47], DBDA [48], DBMA [49], DSGSF [50], and AMGCFN [51]. These methods cover unsupervised learning and supervised learning algorithms with different architectures, such as 1D-CNN, 2D-CNN, 3D-CNN, autoencoder, and attention mechanism network. The architectures and hyperparameters of the above-mentioned comparative models are described in the corresponding published papers.

The classification results of the IP dataset are demonstrated in Table 5. From Table 5, it can be seen that the 1D-CNN achieves the worst results with only 75.67% OA. The main reason is considered that the spatial information is not utilized for classification. Compared with the 1D-CNN, the 2D-CNN, 3D-CNN, and 3D-CAE improve the OA to 83.48%, 84.08%, and 82.27%, respectively, because they consider both spectral and spatial information simultaneously. The accuracies of DBDA and DBMA are improved further by introducing attention mechanism and dense structure. For L3DDAN, the highest classification accuracy is achieved thanks to the autoencoder structure and spectral–spatial joint dense block.

**Figure 9:**
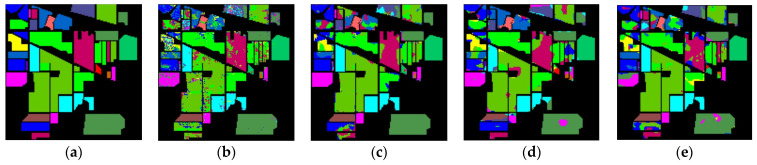

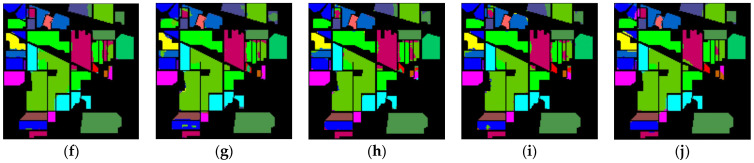
Classification maps of different methods for the IP: (**a**) ground-truth; (**b**) 1D-CNN; (**c**) 2D-CNN; (**d**) 3D-CNN; (**e**) 3D-CAE; (**f**) DBDA; (**g**) DBMA; (**h**) DSGSF; (**i**) AMGCFN; (**j**) L3DDAN.

The classification maps of different methods for IP datasets are shown in Figure 9. It can be observed that there are a large amount of misclassified pixels in (b), (c), (d) of Figure 9. In contrast, the classification map of L3DDAN is the most similar to the ground-truth.

The classification results of the UP dataset are reported in Table 6. From Table 6, it can be seen that the L3DDAN achieved the best performance on OA (99.31%), AA (98.67%), Kappa (0.9908), and 6 of 9 specific classes. The worst result is achieved by 1D-CNN and the results of DBDA and DBMA are similar.

**Figure 10:**
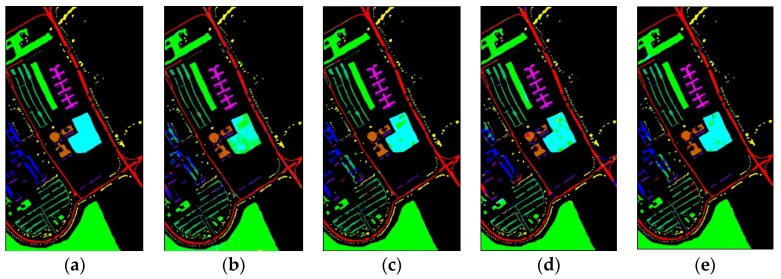

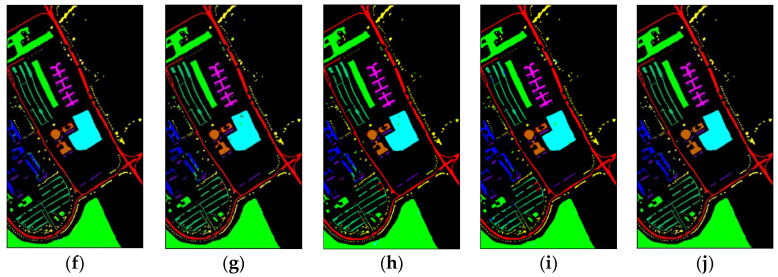
Classification maps of different methods for the UP: (**a**) ground-truth; (**b**) 1D-CNN; (**c**) 2D-CNN; (**d**) 3D-CNN; (**e**) 3D-CAE; (**f**) DBDA; (**g**) DBMA; (**h**) DSGSF; (**i**) AMGCFN; (**j**) L3DDAN.

Figure 10 shows classification maps of different methods for the UP dataset. More misclassified pixels appear in some classes with few labeled pixels such as Gravel and Bare Soil.

The categorized results for SV dataset are demonstrated in Table 7. From Table 7, it can be seen that the L3DDAN obtains the best results with 99.64% OA, 99.69% AA, and 0.9960 Kappa.

**Figure 11:**
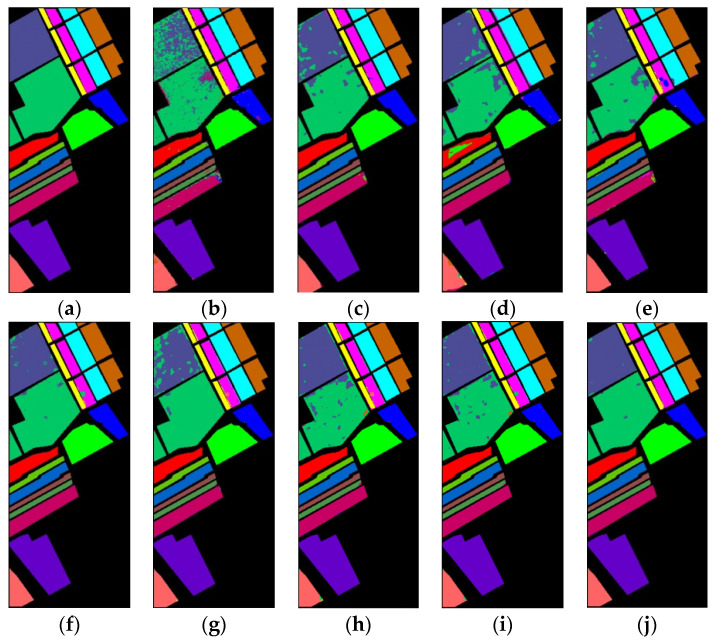
Classification maps of different methods for the SV: (**a**) ground-truth; (**b**) 1D-CNN; (**c**) 2D-CNN; (**d**) 3D-CNN; (**e**) 3D-CAE; (**f**) DBDA; (**g**) DBMA; (**h**) DSGSF; (**i**) AMGCFN; (**j**) L3DDAN.

The classification maps for SV dataset are shown in Figure 11. There are obvious mislabeled pixels in areas of Vineyard—untrained and Grapes—untrained in classification maps of 1D-CNN, 2D-CNN, and DBMA. The classification map of the proposed method shows less mislabeled pixels than other methods.

#### 3.3.2. Impact of Training Sample Size

**Figure 12:**
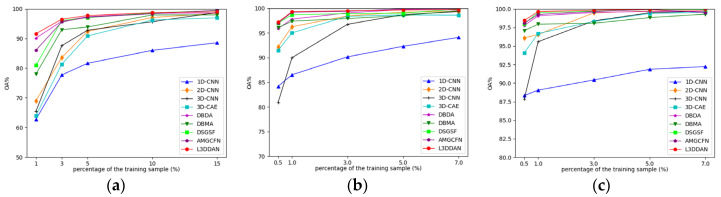
The impact of training samples proportions on classification accuracy for all datasets: (**a**) IP; (**b**) UP; (**c**) SV.

Due to the limited number of HRSI labeled samples, the classification performance with a small number of training samples becomes particularly important. A series of experiments were conducted to explore the OA variations with different proportions of training samples for all methods. For the IP dataset, the training samples proportion is set to 1%, 3%, 5%, 10%, and 15%, and for the UP and SV datasets, they are set to 0.5%, 1%, 3%, 5%, and 7%. The experimental results are shown in Figure 12.

From Figure 12, the classification accuracy of 1D-CNN is the lowest in most cases and the increase in training sample number only improves limited OA. The reason is considered that the spatial features are discarded and the feature extraction capability of 1D-CNN is insufficient. The classification accuracies of other methods are close when the proportion of training samples is greater than 10% for the IP dataset and 3% for the UP and SV datasets. As the training samples proportion decreased, declines of all classification accuracies with different levels occurred. The proposed L3DDAN maintains the highest classification accuracies for all datasets. It indicates that the L3DDAN can extract more distinguishable features from limited training samples for classification.

#### 3.3.3. Comparison of Parameter Quantity

The number of trainable parameters is an important indicator for evaluating the model. Table 8 lists the numbers of trainable parameters for all methods.

For all datasets, the number of trainable parameters of proposed L3DDAN is the second smallest. For the IP dataset, the trainable parameter number in L3DDAN has been reduced to 4.2%, 0.6%, 30.3%, 44.5%, 27.9%, 38.97%, and 46.23% of those in 2D-CNN, 3D-CNN, 3D-CAE, DBDA, DBMA, DSGSF, and AMGCFN, respectively. For the UP and SV datasets, the trainable parameter numbers of L3DDAN also have been significantly reduced.

### 3.4. Discussion

Based on the results in Table 5, Table 6 and Table 7, it is evident that the classification results of 1D-CNN (75.67% for IP, 86.25% for UP, and 89.19% for SV) are the lowest on all datasets. This indicates that the feature extraction ability of 1D convolution kernels is insufficient for HRSI, because it can only extract spectral features. By introduction of spatial features, the classification accuracies of 2D-CNN (83.48% for IP, 95.31% for UP, and 97.17% for SV) and 3D-CNN models (92.26% for IP, 92.26% for UP, and 94.42% for SV) have been significantly improved. It is notable that the classification results of 3D-CNN are lower than those of 2D-CNN on all datasets. This indicates that the feature extraction ability of models based on convolution operations for HRSI classification not only relates to the kernel type, but also to the model architecture. The classification accuracies of 3D-CAE (95.20% for UP and 96.12% for SV) are further improved by introducing the SAE into 3D-CNN. This indicates that the deep features can be learned by unlabeled data. Other compared methods significantly improved feature extraction capabilities by introducing a multi-branch structure. In DBDA and DBMA, both spatial and spectral branches are based on the attention mechanism. The difference between the two models is that the two branches are parallel in DBDA and serial in DBMA. In proposed L3DDAN, the two separated branches are replaced by a spatial–spectral joint branch to extract deep spatial–spectral joint features in HRSI. The classification accuracies of L3DDAN (97.65% for IP, 99.31% for UP, and 99.64% for SV) are higher than those of DBDA (94.97% for IP, 98.65% for UP, and 98.35% for SV) and DBMA (94.76% for IP, 98.62% for UP, and 97.57% for SV).

The experimental results of Figure 12 indicate that all methods except 1D-CNN can achieve satisfactory classification accuracies when there are sufficient training samples. As the training sample proportion decreases, the classification accuracies of 2D-CNN, 3D-CNN, and 3D-CAE decrease more significantly, even below that of 1D-CNN for UP and SV. Based on Table 8, the main reason for the above-mentioned results is that there are too many trainable parameters in 2D-CNN (4,013,386) and 3D-CNN (30,536,176) than 1D-CNN (72,216). The trainable parameter numbers of DBDA (382,326), DBMA (609,791), DUGS (436,791), and AMGCFN (368,217) are far fewer than 2D-CNN and 3D-CNN and these models can achieve better classification accuracies. The number of trainable parameters in L3DDAN is further reduced by the introduction of the spatial–spectral joint branch and it maintains the highest classification accuracies with the minimum number of training samples.

## 4. Conclusions

In this paper, a Lightweight 3D Dense Autoencoder Network is proposed for HRSI classification. The framework of L3DDAN is designed as an SAE to utilize unlabeled samples by unsupervised learning. In addition, the Spatial–Spectral Joint Dense Block is introduced to replace the traditional separated spatial and spectral feature extraction blocks. This architecture not only improves the spatial–spectral joint features extraction ability of L3DDAN, but also reduces the number of trainable parameters. Extensive experiments’ results demonstrate that the L3DDAN surpasses eight different framework state-of-the-art methods in the classification accuracies with a small number of training samples. In addition, the L3DDAN can still maintain excellent deep feature extraction ability when the number of training samples is decreased. This is extremely important for HRSI classification with limited labeled samples.

However, the introduction of SAE makes a significant increase in training time, because all samples are used for the training of the encoder and decoder. The training time of the proposed L3DDAN is greater than other methods except 3D-CAE. This process consumes significant computational resources. In addition, the generalizability of L3DDAN on more HRSI datasets still needs to be verified. The future directions of our work mainly focus on the above-mentioned two limitations: (1) The architecture of SAE will be optimized to reduce the time consumption; (2) The parameters will be optimized to make L3DDAN exhibit state-of-the-art performance on more datasets for application.

## Figures and Tables

**Table 1:** Network structure of L3DDAN.

Layer		Kernel Size	Strides
Encoder	3D Convolution	20@(1,1,7) ^1^	(1,1,3)
	3D Convolution	10@(1,1,7)	(1,1,1)
	3D Convolution	10@(1,1,7)	(1,1,1)
	3D Convolution	10@(1,1,7)	(1,1,1)
	3D Convolution	50@(1,1,65)	(1,1,1)
Decoder	3D Convolution	100@(3,3,1)	(1,1,1)
	3D Convolution	200@(3,3,1)	(1,1,1)
Classifier	3D pooling	-	-
	Full connection	60@	-

^1^ The value of 20 and (1,1,7) represents the number and size of kernel, respectively.

**Table 2:** The number of training, validation, and test samples in IP dataset.

Order	Class	Total Number	Train	Validation	Test
1	Alfalfa	46	3	3	40
2	Corn no-till	1428	42	42	1344
3	Corn min-till	830	24	24	782
4	Corn	237	7	7	223
5	Grass–pasture	483	14	14	455
6	Grass–trees	730	21	21	688
7	Grass–pasture–mowed	28	3	3	22
8	Hay—windrowed	478	14	14	450
9	Oats	20	3	3	14
10	Soybean no-till	972	29	29	914
11	Soybean min-till	2455	73	73	2309
12	Soybean—clean	593	17	17	559
13	Wheat	205	6	6	193
14	Woods	1265	37	37	1191
15	Buildings–Grass–Trees–Drives	386	11	11	364
16	Stone–Steel–Towers	93	3	3	87
	Total	10,249	307	307	9635

**Table 3:** The number of training, validation, and test samples in UP dataset.

Order	Class	Total Number	Train	Validation	Test
1	Asphalt	6631	66	66	6499
2	Meadows	18,649	186	186	18,277
3	Gravel	2099	20	20	2059
4	Trees	3064	30	30	3004
5	Painted metal sheets	1345	13	13	1319
6	Bare Soil	5029	50	50	4929
7	Bitumen	1330	13	13	1304
8	Self-Blocking Bricks	3682	36	36	3610
9	Shadows	947	9	9	929
	Total	42,776	423	423	41,930

**Table 4:** The number of training, validation, and test samples in SV dataset.

Order	Class	Total Number	Train	Validation	Test
1	Broccoli–green–weeds_1	2009	20	20	1969
2	Broccoli–green–weeds_2	3726	37	37	3652
3	Fallow	1976	19	19	1938
4	Fallow–rough–plow	1394	13	13	1368
5	Fallow—smooth	2678	26	26	2626
6	Stubble	3959	39	39	3881
7	Celery	3579	35	35	3509
8	Grapes—untrained	11,271	112	112	11,047
9	Soil-vineyard—develop	6203	62	62	6079
10	Corn-senesced–green–weeds	3278	32	32	3214
11	Lettuce—romaine—4 wk	1068	10	10	1048
12	Lettuce—romaine—5 wk	1927	19	19	1889
13	Lettuce—romaine—6 wk	916	9	9	898
14	Lettuce—romaine—7 wk	1070	10	10	1050
15	Vineyard—untrained	7268	72	72	7124
16	Vineyard—vertical trellis	1807	18	18	1771
	Total	54,129	533	533	53,063

**Table 5:** Classification results (%) for IP dataset with 3% training samples.

Class	1D-CNN	2D-CNN	3D-CNN	3D-CAE	DBDA	DBMA	DSGSF	AMGCFN	L3DDAN
1	100	100	87.50	100	100	73.47	100	90.43	100
2	65.56	71.92	75.51	78.64	94.47	94.78	96.08	94.20	97.76
3	72.26	86.97	84.54	74.63	96.55	97.13	87.08	95.72	94.30
4	48.60	72.32	90.15	64.10	88.14	97.27	96.31	96.16	93.39
5	83.41	90.56	82.80	82.48	93.42	100	99.11	90.22	99.76
6	84.87	95.38	88.99	89.10	95.25	99.27	99.42	97.23	99.42
7	51.52	100	36.00	75.00	70.97	53.66	76.19	91.70	62.86
8	88.17	88.05	99.78	92.80	100	98.47	100	99.43	100
9	100	100	44.00	100	65.00	38.24	50.00	99.44	66.67
10	72.34	71.69	69.15	82.40	98.50	98.71	93.43	93.25	97.80
11	76.75	86.42	87.79	88.74	94.65	92.74	98.10	97.13	99.04
12	57.36	72.25	77.90	59.41	92.04	87.69	92.23	90.64	95.77
13	92.75	100	98.32	92.49	99.48	100	95.98	97.53	100
14	90.29	94.05	91.08	87.95	97.56	98.12	98.05	98.93	97.78
15	63.81	75.41	90.83	85.03	84.72	86.02	98.39	91.37	96.73
16	89.01	91.75	87.65	85.37	95.56	100	88.04	91.17	93.55
OA	75.67	83.48	84.08	83.20	94.97	94.76	96.02	95.55	97.65
AA	68.31	69.88	77.96	76.17	95.29	93.64	91.77	94.92	97.62
Kappa	0.7222	0.8113	0.8176	0.8080	0.9426	0.9401	0.9545	0.9492	0.9733

**Table 6:** Classification results (%) for UP dataset with 1% training samples.

Class	1D-CNN	2D-CNN	3D-CNN	3D-CAE	DBDA	DBMA	DSGSF	AMGCFN	L3DDAN
1	91.38	96.72	89.96	92.20	99.29	99.26	97.83	99.23	99.49
2	88.53	96.22	96.32	97.48	99.73	99.44	99.71	99.97	99.86
3	73.04	94.77	78.66	88.62	95.93	93.99	99.60	98.79	98.30
4	90.87	99.19	94.12	99.27	99.65	98.59	99.66	95.49	97.44
5	99.33	100	97.33	99.18	99.77	98.73	99.92	99.83	99.92
6	81.45	98.72	88.23	96.15	99.60	99.88	99.82	99.99	99.98
7	66.36	99.77	77.43	98.81	98.78	100	96.95	99.79	99.62
8	76.26	79.58	91.21	84.89	91.67	94.15	92.74	98.94	97.00
9	98.94	99.45	88.09	99.40	97.47	96.75	97.76	91.66	99.34
OA	86.25	95.31	92.26	95.20	98.65	98.62	98.68	99.19	99.31
AA	84.76	92.88	88.07	91.39	97.57	97.45	98.22	98.19	98.67
Kappa	0.8156	0.9374	0.8972	0.9360	0.9821	0.9817	0.9825	0.9893	0.9908

**Table 7:** Classification results (%) for SV dataset with 1% training samples.

Class	1D-CNN	2D-CNN	3D-CNN	3D-CAE	DBDA	DBMA	DSGSF	AMGCFN	L3DDAN
1	99.79	100	97.24	99.00	100	100	100	99.99	100
2	98.03	99.97	90.74	99.89	100	99.86	100	99.99	100
3	90.17	98.69	98.40	93.44	99.64	99.64	100	100	99.95
4	98.75	98.98	96.15	96.19	96.33	92.74	98.75	98.72	98.98
5	96.44	97.98	91.33	92.60	99.92	96.81	99.22	98.39	99.32
6	99.92	99.97	99.65	99.95	100	100	99.95	99.91	99.97
7	99.38	99.41	98.33	100	100	100	100	99.97	100
8	81.08	93.58	92.29	96.28	96.43	93.55	99.17	98.39	99.98
9	98.97	99.63	99.50	98.81	100	100	99.80	100	99.85
10	90.19	98.38	96.75	98.19	98.80	96.74	98.66	99.11	99.78
11	92.49	96.67	99.32	91.62	100	99.90	98.68	99.49	99.71
12	96.79	98.65	98.81	99.68	100	97.93	99.79	99.93	100
13	95.14	99.89	94.79	96.18	99.65	98.77	98.67	99.22	99.56
14	96.34	98.68	99.81	98.12	95.32	97.54	97.63	98.66	96.85
15	65.46	92.56	85.21	87.31	95.35	97.75	99.75	98.79	98.62
16	98.37	100	100	99.83	100	99.43	100	99.76	100
OA	89.19	97.17	94.42	96.12	98.35	97.57	99.51	99.26	99.64
AA	93.76	98.42	95.68	97.53	98.84	98.12	99.38	99.39	99.69
Kappa	0.8799	0.9685	0.9379	0.9568	0.9816	0.9729	0.9946	0.9918	0.9960

**Table 8:** The number of trainable parameters for all methods.

	1D-CNN	2D-CNN	3D-CNN	3D-CAE	DBDA	DBMA	DSGSF	AMGCFN	L3DDAN
IP	72,216	4,013,386	30,536,176	561,472 ^1^	382,326	609,791	436,791	368,217	170,236 ^1^
UP	63,249	4,012,119	5,860,841	561,017 ^1^	202,751	324,376	423,383	343,614	89,879 ^1^
SV	74,216	4,013,386	6,076,656	561,472 ^1^	389,622	621,407	437,099	369,009	170,236 ^1^

^1^ The parameters in decoders have not been considered, because the decoders are not used for classification.

## Data Availability

All datasets used in this research are open accessible online (http://www.ehu.eus/ccwintco/index.php?title=Hyperspectral_Remote_Sensing_Scenes, accessed on 15 October 2023).

## Abbreviations

The following abbreviations are used in this manuscript:

HRSI	Hyperspectral Remote Sensing Image
PCA	Principal Component Analysis
MAP	Morphological Attribute Profiles
DL	Deep Learning
SAE	Stacked Autoencoder
3DDRN	3D Deep Residual Network
CNN	Convolutional Neural Network
CS2ADN	Cooperative Spectral–Spatial Attention Network
L3DDAN	Lightweight 3D Dense Autoencoder Network
S^2^DB	Spectral–Spatial Joint Dense Block
S^2^RB	Spectral–Spatial Joint Residual Block
IP	Indian Pines
AVIRIS	Airborne Visible Infrared Imaging Spectrometer
UP	University of Pavia
ROSIS	Reflective Optics System Imaging Spectrometer
SV	Salinas Valley
OA	Overall Accuracy
AA	Average Accuracy
FE	Feature Extraction
